# Hydration behaviors, knowledge, and attitudes among Chinese recreational marathon runners: a cross-sectional study

**DOI:** 10.3389/fnut.2025.1621966

**Published:** 2025-08-13

**Authors:** Jingyi Zhang, Yan Chen, Xueyuan Zhao, Xiangxin Li, Wei Wen, Wenqiang Wu, Menghui Zou, Junqiang Qiu

**Affiliations:** ^1^School of Education, Beijing Sport University, Beijing, China; ^2^Exercise Science School, Beijing Sport University, Beijing, China; ^3^Beijing Sports Nutrition Engineering Research Center, Beijing, China

**Keywords:** marathon, recreational runners, rehydration, hydration practices, hydration knowledge

## Abstract

**Objectives:**

This cross-sectional study aimed to assess fluid replenishment behaviors, knowledge, and attitudes among Chinese recreational marathon runners and to inform future health education strategies regarding optimal hydration.

**Design:**

The survey was conducted between January and December 2024. Based on previously validated questionnaires, the instrument was adapted and administered to assess hydration behaviors, knowledge, and attitudes among marathon participants in major road races across China. A total of 461 valid responses were collected, including 357 male participants (38.9 ± 9.3 years) and 104 female participants (39.5 ± 8.9 years), representing a range of athletic performance levels. The response rate was 94.9%. Data were analyzed using IBM SPSS 27.0 and Microsoft Excel.

**Result:**

Over 70% of runners (72.5%) failed to meet recommended daily fluid intake standards (*p* < 0.001). Over half (57.7%) consumed 150 mL of fluid before the race, mainly plain water before (68.1%) and sports drinks during (56.6%) and after (54.0%) the race. Participants primarily obtained hydration knowledge from friends/family (54.9%) and social media (45.6%). Most participants (93.7%) acknowledged water’s health benefits and were willing (92.2%) to improve hydration habits to enhance athletic performance (*p* < 0.001 for all). Hydration knowledge varied by gender, age, and training level. Participants better understood pre- and mid-exercise hydration and the concept that thirst indicates existing dehydration. However, only 21.5% knew the recommended daily intake (1500–1700 ml), 39.9% understood the differences among mineral, spring, and purified water, and 46.8% recognized the need for post-exercise rehydration.

**Conclusion:**

These findings indicate that over half of recreational marathon runners are at risk of dehydration, highlighting that a lack of knowledge poses a dilemma for some runners who fall into a dehydrated state. This underscores the need for targeted education and awareness programs on scientific hydration knowledge and attitudes among recreational marathon runners to improve their health and athletic performance.

## 1 Introduction

In China, running has witnessed rapid growth as a popular endurance sport, driven by the continued implementation of the National Fitness Strategy and the booming development of road running events. According to data released by the Chinese Athletics Association, over 700 road races were held across the country in 2024, attracting more than 7 million participants (1).

Rehydration is critical to human health and athletic performance (2), especially for marathon athletes. As a typical long-distance endurance sport, marathons often expose athletes to extreme climatic conditions, such as high temperatures and humidity, for extended periods during training and competition. These conditions substantially increase sweat loss, leading to considerable water and electrolyte depletion (3). Inadequate or untimely rehydration may predispose athletes to hydration-related illnesses (4, 5). Therefore, maintaining stable hydration during marathons is not only key to sustaining athletic performance but also an essential safeguard against health risks.

However, for marathon runners, maintaining dynamic hydration balance—the ongoing regulation and fluctuation between fluid intake and sweat loss (6)—during daily training and competition presents multiple physiological challenges, primarily due to the effects of persistent sweat loss and electrolyte depletion on plasma volume stability and intracellular fluid balance (7). This process is highly susceptible to dysregulation due to individual differences in athletes’ responses to environmental temperature, humidity, and exercise intensity (8), which can affect their health and performance. Existing studies indicate that the oxygen uptake of runners of varying levels during marathon exercise ranges from 60 to 86% of VO₂ max (9), and the hourly sweating rate of adult participants can reach 1.2 to 2.5 L/h. This is accompanied by significant electrolyte loss, with sodium ion (Na+) loss reaching up to 20–80 mmol/L (10). Water and Na + deficiency are considered risk factors for exercise heat stroke (11, 12), exertional heat exhaustion, and exertional heat cramps (13). Dehydration impairs physiological functions, leading to decreased athletic performance (14–16), excessive consumption of plain water without electrolyte supplementation may dilute blood sodium concentration and induce symptoms of hyponatremia (17).

Currently, the number of marathons organized and the number of participants in health promotion-oriented marathons in China are increasing (18). However, systematic studies on the attitudes, knowledge, and behaviors regarding rehydration among Chinese recreational marathon runners remain relatively scarce. Although some research on sports rehydration has been conducted, most studies focus on professional athletes and college students, with fewer addressing Chinese recreational marathon runners (19–23). Compared to professional athletes, recreational marathon runners differ significantly in exercise level, training mode, and physical condition (24); their rehydration needs and behavioral characteristics also vary. Existing studies suggest that the post-race fluid loss of recreational marathon runners who finish at a low intensity is generally below the physiological critical threshold of 2% of body weight (25). However, under conditions of high temperature (above 30°C), high humidity (relative humidity above 70%), or complex terrain, their sweat loss rate may increase non-linearly (7), potentially exceeding the 2% body weight threshold affecting exercise performance. This can result in an increased cardiovascular load and compensatory dysregulation of thermoregulatory functions, presenting potential risks (26).

Therefore, the aim of this study is to investigate the daily hydration behavior of recreational marathon runners in China, to understand their hydration practices and strategies during running, and to analyze the influence of factors such as gender, age, and performance level on their hydration behavior, knowledge, and attitudes. The findings aim to provide a theoretical basis for developing evidence-based hydration guidelines, optimizing health risk management, and enhancing athletic performance.

## 2 Materials and methods

### 2.1 Subjects

This study was conducted on long-distance runners participating in various marathons, including full marathons, half marathons, and other related events. The study adopted a convenience sampling method, and participants were recruited through four channels- running groups, community organizations, sports associations, and the Internet to ensure a broad and representative sample. Individuals who met the following inclusion criteria were accepted into the study: (a) participants aged 18 years or older; (b) required to have completed at least one full or half marathon within the past 6 months; and (c) in good health and free of serious illness. Participants were excluded from the study if they: (a) had serious cardio-vascular or cerebrovascular diseases; (b) had abnormalities in liver and kidney functions; (c) used medications that may affect nutrition or metabolism (e.g., antibiotics, antidepressants, hypoglycemic agents, etc.); (d) were pregnant or lactating; and (e) had experienced a significant change in body weight in the past 3 months. The survey began with a detailed pre-survey statement outlining the objectives of the study, data confidentiality procedures, and a brief written explanation to assist participants in accurately understanding and answering the questions. After fully comprehending the study content, participants were required to give verbal consent in accordance with the principle of informed consent. This study received ethical approval from the Institutional Review Board of the School of Exercise Science at Beijing Sport University, on February 1, 2024 (Approval Number: 2024256H).

The survey population includes a variety of genders, ages, and sports levels. Participants were divided by age into five groups (18–34, 35–44, 45–54, 55–64, and ≥65 years). Performance levels were categorized according to the official grading system based on self-reported personal best times across different age and sex groups, following the Chinese Athletics Association Public Road Running Skill Level Standards (2023) (27). These standards classify runners into Public Elite, Public Level 1 (L1), and Public Level 2 (L2) based on gender and age, reflecting the athletic ability and experience of participants at different levels.

### 2.2 Survey procedures

This study was a cross-sectional survey. The structured questionnaire used as the primary research instrument was developed based on relevant literature and adapted from two pre-validated questionnaires by Zhao et al. and Zhang et al. (20, 28), which have been widely applied among the general Chinese population and athletes. The questionnaire was reviewed by seven experts in the fields of sports nutrition, exercise science, and public health. The experts evaluated each item in terms of clarity, content coverage, and applicability. Following the expert review, a pilot test was conducted among approximately 50 amateur marathon runners to assess the comprehensibility and logical structure of the items. Based on the feedback from both the experts and the pilot participants, revisions were made to the wording, structural layout, and response options to ensure that the items were scientifically sound, logically coherent, and possessed strong practical relevance and specificity. The questionnaire covered several dimensions closely related to the hydration status and health behaviors of marathon participants. The final questionnaire consisted of 35 items, divided into four major sections: basic information (10 items), hydration behavior (11 items), hydration knowledge (10 items), and hydration attitude (4 items). Knowledge-based items were designed in accordance with the latest scientific perspectives and expert recommendations. These procedures were implemented to enhance the face validity and usability of the questionnaire, ensuring its suitability for large-scale data collection. These dimensions included basic information (demographic characteristics such as gender, age, height, weight, etc.), rehydration behaviors (including daily rehydration practices and behaviors during running), knowledge of rehydration (daily water intake, rehydration methods, the distinction between rehydration and water intake, and signs of dehydration, etc.), and attitudes toward rehydration (the importance of rehydration for health, willingness to change rehydration habits, and sources of knowledge regarding rehydration, etc.).

The survey was conducted between January and December 2024. Online distribution was carried out via official accounts on platforms such as the Chinese Athletics Association’s official website, marathon event websites, marathon registration applications, and social media platforms, reaching a broad range of participants. Offline distribution and completion were conducted on-site at marathon events such as the Beijing Marathon, Tianjin Marathon, and Guangzhou Marathon. All participants completed the questionnaire independently after receiving brief instructions, to ensure the authenticity and completeness of the responses. A total of 486 questionnaires were collected, and after strict screening, 461 valid questionnaires were obtained, resulting in a validity rate of 94.9%. The samples included participants of varying genders, ages, and performance levels, providing a representative and comprehensive profile of the current characteristics of rehydration behavior among Chinese recreational marathon runners.

### 2.3 Statistical analysis

Questionnaire data were statistically analyzed using IBM SPSS 27.0 and Microsoft Excel. Continuous variables (e.g., demographic characteristics) were expressed as means ± standard deviations (Mean ± SD), while categorical variables were expressed as frequencies and percentages. The Shapiro–Wilk test was first performed to assess the normality of continuous variables. For between-group comparisons, if the data met the assumption of normality, the independent samples t-test was applied; otherwise, the non-parametric Mann–Whitney U test was used. Associations between categorical variables (e.g., gender, age group, performance level) were examined using the chi-square (χ^2^) test. For binary proportion data, a one-sample binomial test was used to determine whether the observed proportion significantly differed from a theoretical value (e.g., 50%). All tests were two-tailed, and statistical significance was defined as *p* < 0.05.

## 3 Results

### 3.1 Demographics and running-related information of recreational marathon runners

There were 357 male participants (77.4%) and 104 female participants (22.6%) in this survey. The age range of the majority of the participants was 35–44 years (41.6%) (Table 1). The final sample did not include any participants aged 65 years or older; therefore, the highest age group analyzed was 55–64 years. The mean height was 173.6 ± 6.1 cm for males and 161.9 ± 4.8 cm for females, while the weight averaged 70.0 ± 8.5 kg for males and 55.4 ± 6.2 kg for females, with males significantly taller and heavier than females (*p* < 0.001). BMI was classified according to the Health Industry Standard of the People’s Republic of China (WS/T 428–2013) (29), with four categories: underweight (BMI < 18.5), normal weight (18.5 ≤ BMI < 24.0), overweight (24.0 ≤ BMI < 28.0), and obesity (BMI > 28.0). The mean body mass index (BMI) of the runners was 22.8 ± 2.4 kg/m^2^. Among them, 67.2% (310) were of normal weight, 26.9% (124) were overweight, 2.6% (12) were obese, and 3.3% (15) were underweight. The mean BMI of male participants was 23.6 ± 2.3 kg/m^2^, which was significantly higher than that of females at 21.5 ± 2.3 kg/m^2^ (*p* < 0.001). In terms of performance level, the largest number of participants was in Public L2, with 169 participants (36.7%), followed by Public L1, which had 128 participants (27.8%). The proportion of females who did not reach the performance level was significantly higher than that of males (35.6% vs. 15.45%; *p* < 0.001). Runners with less than 3 years of running experience were the most prevalent group, comprising 163 runners (35.4%). The monthly running volume was mainly concentrated in the 100–200 km range, with 168 participants (34.9%). The proportion of female participants running less than 20 km per month was significantly higher than that of males (19.2% vs. 9.0%; *p* = 0.004). The largest group of participants, accounting for 43.6%, had participated in 0–5 previous marathons (Table 1).

**Table 1:** Essential information and running-related traits of runners.

Index	Total	Male	Female	*p*
Sample size No. (%)	461(100%)	357(77.4%)	104(22.6%)	<0.001
Age (years)	39.1 ± 9.3	38.9 ± 9.3	39.5 ± 8.9	0.5501
Height (cm)	171.0 ± 7.6	173.6 ± 6.1	161.9 ± 4.8	<0.001
Weight (kg)	66.9 ± 10.0	70.0 ± 8.5	55.4 ± 6.2	<0.001
BMI (kg/m^2^)	22.8 ± 2.4	23.6 ± 2.3	21.5 ± 2.3	<0.001
Age group classification No. (%)				
18–34 years	142(30.8%)	114(31.9%)	28(26.9%)	0.330
35–44 years	192(41.6%)	148(41.5%)	44(42.3%)	0.877
45–54 years	101(21.9%)	74(20.7%)	27(26.0%)	0.256
55–64 years	26(5.6%)	21(5.9%)	5(4.8%)	0.676
BMI classification No. (%)				
Underweight	15(3.3%)	3(0.8%)	12(11.5%)	<0.001
Normal weight	310(67.2%)	231(64.7%)	79(76.0%)	0.031
Overweight	124(26.9%)	111(31.1%)	13(12.5%)	<0.001
Obesity	12(2.6%)	12(3.4%)	0(0.0%)	0.058
Performance levels No. (%)				
Public Elite	11(10.6)	61(17.1)	72(15.6)	0.108
Public Level 1	20(19.2)	108(30.3)	128(27.8)	0.027
Public Level 2	36(34.6)	133(37.3)	169(36.7)	0.623
Not certified	37(35.6)	55(15.4)	92(20.0)	<0.001
Running experience No. (%)				
0–3 years	163(35.4%)	119(33.3%)	44(42.3%)	0.092
3–5 years	130(28.2%)	105(29.4%)	25(24.0%)	0.284
5–7 years	71(15.4%)	57(16.0%)	14(13.5%)	0.533
7 years	97(21.0%)	76(21.3%)	21(20.2%)	0.809
Monthly running volume No. (%)				
Under 20 KM	52(11.3%)	32(9.0%)	20(19.2%)	0.004
20–50 KM	35(7.6%)	24(6.7%)	11(10.6%)	0.192
50–100 KM	67(14.5%)	48(13.4%)	19(18.3%)	0.219
100–200 KM	161(34.9%)	130(36.4%)	31(29.8%)	0.214
200–300 KM	108(23.4%)	89(24.9%)	19(18.3%)	0.158
300–500 KM	35(7.6%)	32(9.0%)	3(2.9%)	0.039
Over 500 KM	3(0.7%)	2(0.6%)	1(1.0%)	0.654
Number of marathon participations No. (%)				
0–5	201(43.6%)	150(42.0%)	51(49.0%)	0.204
6–10	115(24.9%)	91(25.5%)	24(23.1%)	0.617
11–15	63(13.7%)	48(13.4%)	15(14.4%)	0.798
16–20	27(5.9%)	23(6.4%)	4(3.8%)	0.321
20–30	21(4.6%)	18(5.0%)	3(2.9%)	0.353
Over 30 times	34(7.4%)	27(7.6%)	7(6.7%)	0.775

### 3.2 Daily hydration practices among Chinese recreational marathon runners

**Figure 1:**
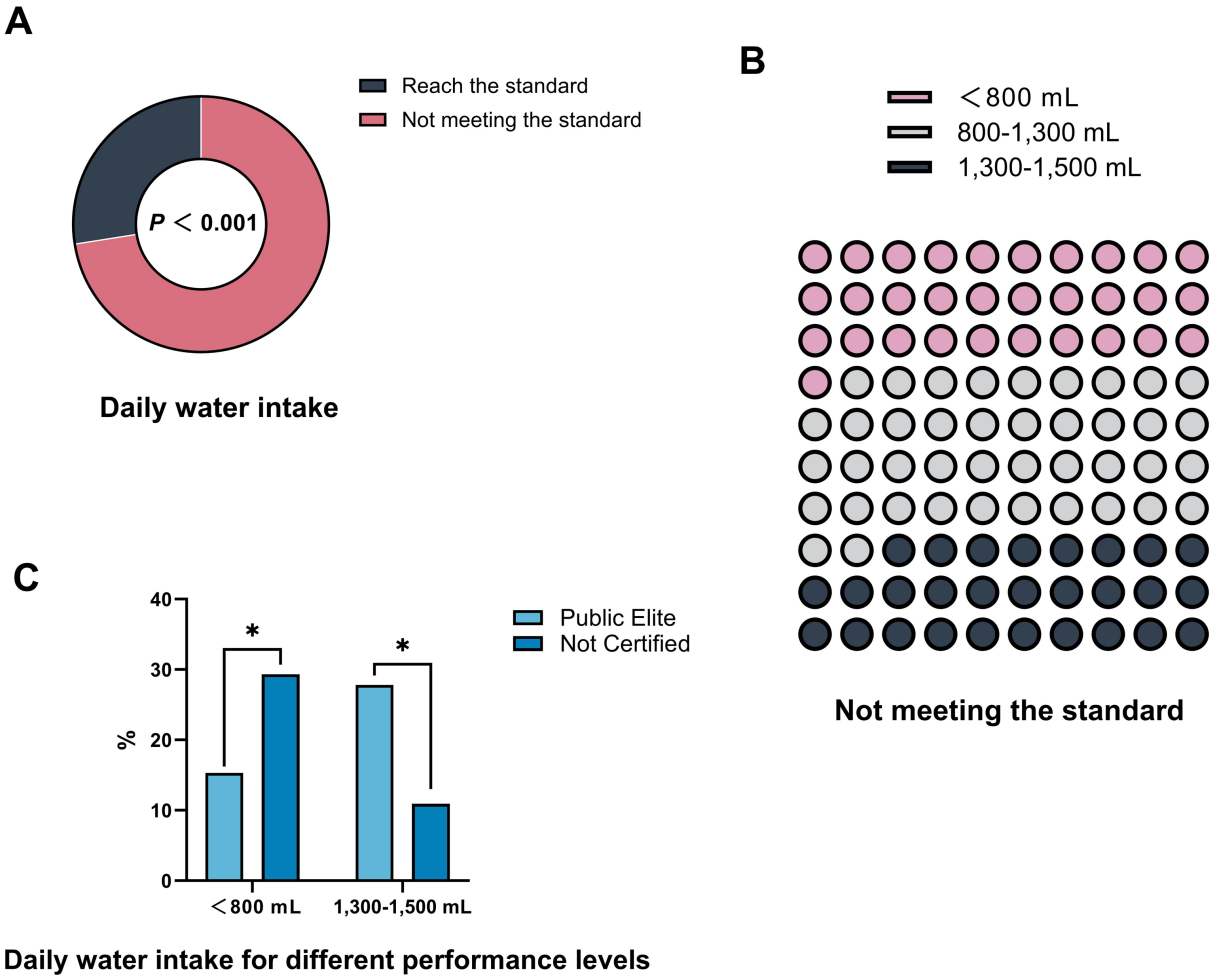
Daily water intake status and subgroup comparisons among Chinese recreational marathon runners. **(A)** Proportion of participants who met (dark gray) versus did not meet (pink) the recommended daily water intake. The difference was statistically significant (*p* < 0.001). **(B)** Distribution of daily water intake levels among participants who did not meet the standard. Intake was categorized as <800 mL (pink), 800–1,300 mL (gray), and 1,300–1,500 mL (dark blue). **(C)** Comparison of daily water intake between “Public Elite” runners and “Not certified” runners. A higher proportion of “Public Elite” runners consumed 1,300–1,500 mL daily, while a greater proportion of “Not certified” runners consumed <800 mL. *p < 0.05.

Figure 1A shows the distribution of individuals who met the recommended daily water intake (27.5%) versus those who did not (72.5%) (*p* < 0.001), according to the *Dietary Guidelines for Chinese Residents* (30), which recommends a daily water intake of 1,500–1700 mL for healthy adults. Notably, among those with insufficient water intake, 22.3% of runners consumed less than 800 mL of water per day (Figure 1B). When analyzed in terms of performance levels, the percentage of those drinking less than 800 mL per day decreased with increasing performance levels. Notably, the proportion of people who consumed less than 800 mL per day was significantly higher among those “Not Certified” than in the “Public Elite” (29.3% vs. 15.3%; *p* = 0.034). In contrast, a significantly higher proportion of individuals in the “Public Elite” drank between 1,300–1,500 mL per day compared to those “Not Certified” (27.8% vs. 10.9%; *p* = 0.005) (Figure 1C).

Drinking timing and habits exhibited similar diversity. 47.9% of participants reported habitual fluid intake regardless of thirst. Among runners of different age groups, those aged 34 and below showed a significantly higher tendency to rely on the sensation of thirst as the primary basis for fluid intake (*p* = 0.002). In contrast, older participants demonstrated more consistent drinking habits than younger participants (*p* = 0.034). Our study indicated that regular drinking habits exhibited an increasing trend with age (*p* = 0.002) (Table 2).

**Table 2:** Daily dietary hydration habits of Chinese recreational marathon runners.

Index	Daily water intake					Timing and pattern of water intake			
	< 800 mL	800-1300 mL	1,300-1500 mL	1,500-1700 mL	> 1700 mL	DLWT	DMWT	SPD	Other
Total No. (%)									
	103(22.3%)	137(29.7%)	94(20.4%)	63(13.7%)	64(13.9%)	73(15.8%)	164(35.6%)	221(47.9%)	3(0.7%)
Gender No. (%)									
Male	81(22.7%)	103(28.9%)	75(21.0%)	51(14.3%)	47(13.2%)	57(16.0%)	128(35.9%)	169(47.3%)	3(0.8%)
Female	22(21.2%)	34(32.7%)	19(18.3%)	12(11.5%)	17(16.3%)	16(15.4%)	36(34.6%)	52(50.0%)	0(0.0%)
*p*	0.741	0.451	0.542	0.473	0.409	0.886	0.816	0.633	0.348
Age No. (%)									
18–34 years	38(26.8%)	45(31.7%)	23(16.2%)	19(13.4%)	17(12.0%)	32(22.5%)	56(39.4%)	53(37.3%)	1(0.7%)
35–44 years	46(24.0%)	58(30.2%)	38(19.8%)	21(10.9%)	29(15.1%)	33(17.2%)	66(34.4%)	92(47.9%)	1(0.5%)
45–54 years	13(12.9%)	29(28.7%)	27(26.7%)	16(15.8%)	16(15.8%)	8(7.9%)	34(33.7%)	58(57.4%)	1(1.0%)
55–64 years	6(23.1%)	5(19.2%)	6(23.1%)	7(26.9%)	2(7.7%)	0(0.0%)	8(30.8%)	18(69.2%)	0(0.0%)
*p*	0.068	0.636	0.241	0.139	0.608	0.002	0.690	0.002	0.939
Performance levels No. (%)									
Public elite	11(15.3%)	20(27.8%)	20(27.8%)^b^	11(15.3%)	10(13.9%)	8(11.1%)	8(11.1%)	22(30.6%)	42(58.3%)
Public Level 1	28(21.9%)	39(30.5%)	29(22.7%)	15(11.7%)	17(13.3%)	16(12.5%)	16(12.5%)	45(35.2%)	66(51.6%)
Public Level 2	38(22.5%)	45(26.6%)	35(20.7%)	24(14.2%)	27(16.0%)	29(17.2%)	29(17.2%)	61(36.1%)	77(45.6%)
Not certified	27(29.3%)^a^	32(34.8%)	10(10.9%)	13(14.1%)	10(10.9%)	20(21.7%)	20(21.7%)	36(39.1%)	36(39.1%)
*p*	0.034	0.559	0.005	0.890	0.716	0.179	0.722	0.071	0.604

^a^*p* = 0.034 vs. “Public Elite”; ^b^*p* = 0.005 vs. “Not Certified” (within group). DLWT: means drinking a large amount of water when feeling thirsty; DMWT means drinking a moderate amount of water when feeling thirsty; SPD means scheduled proactive drinking.

### 3.3 Fluid replacement behavior during exercise

#### 3.3.1 Type of fluid replacement

**Figure 2:**
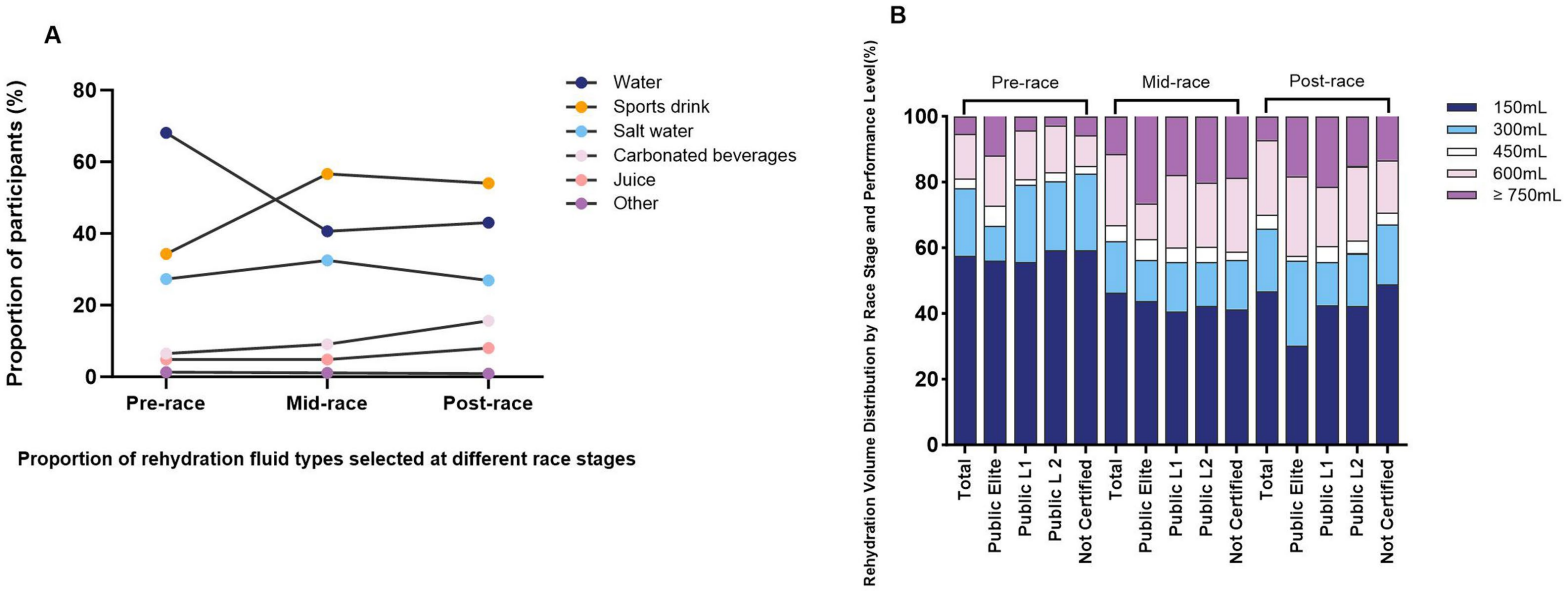
Types and volumes of fluid replacement during training and competition among recreational marathon runners. **(A)** Distribution of fluid type preferences among runners during the race; **(B)** Comparison of in-race fluid intake among runners of different performance levels.

As shown in Figure 2A, sports drinks (56.6%) and water (40.6%) were the most commonly selected types of fluids for rehydration by runners during both training and racing, with a relatively low proportion of other rehydration fluid types being chosen. Runners’ fluid preferences also varied at different stages of the race, with sports drinks as the dominant choice during (56.6%) and after (54.0%) training/racing, while water was the primary choice before (68.1%) training/racing. Notably, 15.6% of participants reported consuming carbonated beverages after the race, with female participants being more inclined to consume carbonated beverages compared to male participants (26.0% vs. 12.6%; *p* < 0.001) (Table 3).

**Table 3:** Types of fluids consumed during races by Chinese recreational marathon runners.

Index	Pre–race					Mid–race					Post–race				
	Water	Salt water	Sports drink	Carbonated beverage	Juice	Water	Salt water	Sports drink	Carbonated beverage	Juice	Water	Salt water	Sports drink	Carbonated beverage	Juice
Total No. (%)															
	314(68.1%)	126(27.3%)	158(34.3%)	30(6.5%)	22(4.8%)	187(40.6%)	150(32.5%)	261(56.6%)	42(9.1%)	22(4.8%)	198(43.0%)	124(26.9%)	249(54.0%)	72(15.6%)	37(8.0%)
Gender No. (%)															
Male	249(69.7%)	98(27.5%)	128(35.9%)	21(5.9%)	16(4.5%)	151(42.3%)	119(33.3%)	202(56.6%)	30(8.4%)	17(4.8%)	154(43.1%)	100(28.0%)	200(56.0%)	45(12.6%)	26(7.3%)
Female	65(62.5%)	28(26.9%)	30(28.8%)	9(8.7%)	6(5.8%)	36(34.6%)	31(29.8%)	59(56.7%)	12(11.5%)	5(4.8%)	44(42.3%)	24(23.1%)	49(47.1%)	27(26.0%)	11(10.6%)
*p*	0.163	0.915	0.185	0.313	0.588	0.160	0.499	0.979	0.979	0.985	0.985	0.318	0.109	<0.001	0.277
Age No. (%)															
18–34	90(63.4%)	30(21.1%)	54(38.0%)	10(7.0%)	6(4.2%)	50(35.2%)	34(23.9%)	82(57.7%)	13(9.2%)	7(4.9%)	48(33.8%)	33(23.2%)	80(56.3%)	24(16.9%)	11(7.7%)
35–44	135(70.3%)	50(26.0%)	65(33.9%)	10(5.2%)	11(5.7%)	82(42.7%)	64(33.3%)	108(56.3%)	19(9.9%)	11(5.7%)	91(47.4%)	53(27.6%)	99(51.6%)	28(14.6%)	15(7.8%)
45–54	73(72.3%)	36(35.6%)	30(29.7%)	9(8.9%)	5(5.0%)	47(46.5%)	40(39.6%)	56(55.4%)	9(8.9%)	4(4.0%)	48(47.5%)	30(29.7%)	55(54.5%)	18(17.8%)	9(8.9%)
55–64	16(61.5%)	10(38.5%)	9(34.6%)	1(3.8%)	0(0.0%)	8(30.8%)	12(46.2%)	15(57.7%)	1(3.8%)	0(0.0%)	11(42.3%)	8(30.8%)	15(57.7%)	2(7.7%)	2(7.7%)
*p*	0.360	0.045	0.606	0.602	0.617	<0.001	0.025	0.985	0.797	0.605	0.064	0.655	0.820	0.584	0.987
Performance levels No. (%)															
Public elite	55(76.4%)	26(36.1%)	24(33.3%)	5(6.9%)	3(4.2%)	34(47.2%)	27(37.5%)	43(59.7%)	8(11.1%)	5(6.9%)	35(48.6%)	19(26.4%)	46(63.9%)	13(18.1%)	4(5.6%)
Public Level 1	85(66.4%)	32(25.0%)	51(39.8%)	10(7.8%)	6(4.7%)	49(38.3%)	37(28.9%)	78(60.9%)	14(10.9%)	5(3.9%)	57(44.5%)	34(26.6%)	73(57.0%)	17(13.3%)	12(9.4%)
Public Level 2	113(66.9%)	44(26.0%)	57(33.7%)	11(6.5%)	6(3.6%)	70(41.4%)	51(30.2%)	97(57.4%)	15(8.9%)	9(5.3%)	74(43.8%)	39(23.1%)	93(55.0%)	28(16.6%)	13(7.7%)
Not certified	61(66.3%)	24(26.1%)	26(28.3%)	4(4.3%)	7(7.6%)	34(37.0%)	35(38.0%)	43(46.7%)	4(4.3%)	3(3.3%)	32(34.8%)	32(34.8%)	37(40.2%)	14(15.2%)	8(8.7%)
*p*	0.440	0.339	0.349	0.780	0.523	0.542	0.351	0.175	0.328	0.674	0.305	0.243	0.015	0.806	0.803

#### 3.3.2 Volume of fluid intake

Overall, runners selected a relatively similar distribution of rehydration volumes before, during, and after the race, with 150 mL and 300 mL being the more common choices, and only a small proportion chose ≥750 mL. As shown in the Table 4, 57.7% of runners reported consuming 150 mL of fluid before the race. In contrast, the proportion of participants choosing a fluid intake of 600 mL increased during the race (21.6%) and after the race (22.6%), which may relate to the runners’ heightened need for hydration and electrolyte replenishment during and following the race (Figure 2B). Notably, participants aged 55 and older in the pre-race phase chose 750 mL of rehydration significantly more often than younger age groups (18.2% vs. 3.9, 5.3, and 4.2%). In the post-race phase, gender analysis showed that female runners were more likely than male runners to consume 150 mL of fluid (*p* = 0.006). In terms of performance level, “Not Certified” runners had a significantly higher proportion of choosing a 150 mL fluid intake compared to “Public Elite” (*p* = 0.023).

**Table 4:** Fluid intake during the race among Chinese recreational marathon runners.

Index	Pre–race					Mid–race					Post–race				
	150 mL	300 mL	450 mL	600 mL	750 mL	150 mL	300 mL	450 mL	600 mL	750 mL	150 mL	300 mL	450 mL	600 mL	750 mL
Total No. (%)															
	239(57.7%)	85(20.5%)	12(2.9%)	56(13.5%)	22(5.3%)	170(46.4%)	57(15.6%)	18(4.9%)	79(21.6%)	42(11.5%)	176(46.8%)	72(19.1%)	16(4.3%)	85(22.6%)	27(7.2%)
Gender No. (%)															
Male	182(57.6%)	70(22.2%)	7(2.2%)	41(13.0%)	16(5.1%)	131(46.5%)	47(16.7%)	12(4.3%)	61(21.6%)	31(11.0%)	124(42.9%)	59(20.4%)	14(4.8%)	70(24.2%)	22(7.6%)
Female	57(58.2%)	15(15.3%)	5(5.1%)	15(15.3%)	6(6.1%)	39(46.4%)	10(11.9%)	6(7.1%)	18(21.4%)	11(13.1%)	52(59.8%)	13(14.9%)	2(2.3%)	15(17.2%)	5(5.7%)
*p*	0.921	0.143	0.137	0.555	0.683	0.997	0.291	0.283	0.996	0.596	0.006	0.255	0.302	0.172	0.555
Age No. (%)															
18–34 years	78(60.9%)	23(18.0%)	3(2.3%)	19(14.8%)	5(3.9%)	50(43.9%)	14(12.3%)	3(2.6%)	27(23.7%)	20(17.5%)	52(45.2%)	26(22.6%)	6(5.2%)	26(22.6%)	5(4.3%)
35–44 years	101(59.8%)	35(20.7%)	2(1.2%)	22(13.0%)	9(5.3%)	82(50.0%)	23(14.0%)	5(3.0%)	28(17.1%)	26(15.9%)	80(52.6%)	25(16.4%)	5(3.3%)	31(20.4%)	11(7.2%)
45–54 years	55(57.9%)	23(24.2%)	3(3.2%)	10(10.5%)	4(4.2%)	36(45.0%)	15(18.8%)	5(6.3%)	20(25.0%)	4(5.0%)	39(44.3%)	17(19.3%)	1(1.1%)	22(25.0%)	9(10.2%)
55–64 years	5(22.7%)	4(18.2%)	4(18.2%)	5(22.7%)	4(18.2%)	2(10.0%)	5(25.0%)	5(25.0%)	4(20.0%)	4(20.0%)	5(25.0%)	4(20.0%)	4(20.0%)	6(30.0%)	1(5.0%)
*p*	0.008	0.710	<0.001	0.466	0.047	0.009	0.359	<0.001	0.422	0.058	0.101	0.657	0.002	0.721	0.422
Performance levels No. (%)															
Public elite	37(56.1%)	7(10.6%)	4(6.1%)	10(15.2%)	8(12.1%)	28(43.8%)	8(12.5%)	4(6.3%)	7(10.9%)	17(26.6%)	20(30.3%)^a^	17(25.8%)	1(1.5%)	16(24.2%)	12(18.2%)
Public Level 1	64(55.7%)	27(23.5%)	2(1.7%)	17(14.8%)	5(4.3%)	46(40.7%)	17(15.0%)	5(4.4%)	25(22.1%)	20(17.7%)	52(42.6%)	16(13.1%)	6(4.9%)	22(18.0%)	26(21.3%)
Public Level 2	87(59.2%)	31(21.1%)	4(2.7%)	21(14.3%)	4(2.7%)	63(42.3%)	20(13.4%)	7(4.7%)	29(19.5%)	30(20.1%)	64(42.4%)	24(15.9%)	6(4.0%)	34(22.5%)	23(15.2%)
Not certified	51(59.3%)	20(23.3%)	2(2.3%)	8(9.3%)	5(5.8%)	33(41.3%)	12(15.0%)	2(2.5%)	18(22.5%)	15(18.8%)	40(48.8%)	15(18.3%)	3(3.7%)	13(15.9%)	11(13.4%)
*p*	0.920	0.171	0.390	0.639	0.040	0.981	0.955	0.746	0.267	0.542	0.152	0.165	0.711	0.477	0.434

^a^*p* = 0.023 vs. “Not Certified” (within group).

**Figure 3:**
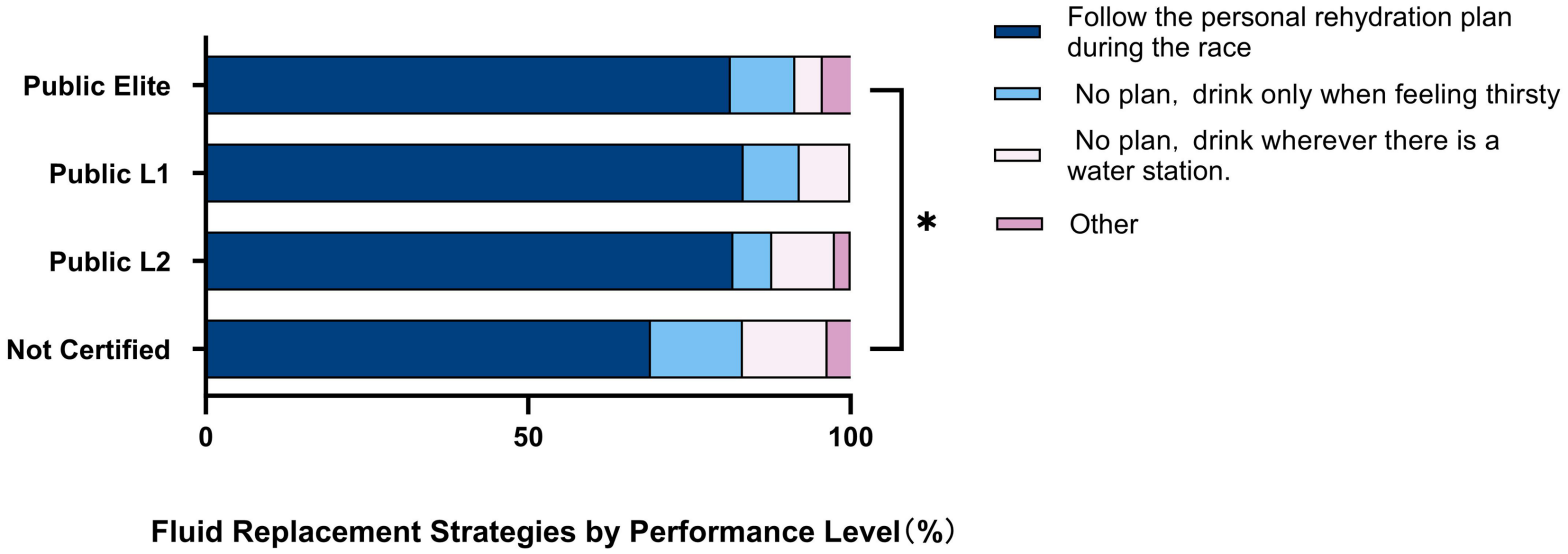
Distribution of fluid replacement strategies during the race across different performance levels among recreational marathon runners. (*) Indicates that there is a significant difference in “Not Certified” compared to other performance levels (*p* = 0.003); FPR means follow the personal rehydration plan made before the race and refill at the rehydration stations according to the set number of kilometers; NPT means have no rehydration plan and refill when they feel thirsty; NPS means no rehydration plan, drink wherever there is a water station.

Figure 3 shows that 77.2% of the participants reported following a pre-race rehydration plan, as well as the distribution of other rehydration strategies. Among runners with different performance levels, those with higher levels are more likely to formulate individualized hydration plans compared to those in the “Not Certified” group (χ^2^ = 13.624, df = 3, *p* = 0.003). Therefore, it can be concluded that the development of pre-race hydration plans is significantly associated with performance levels (Table 5).

**Table 5:** Sources and strategies of fluid intake during races among Chinese recreational marathon runners.

Index	Sources of fluid intake				Hydration strategy			
	Personal supply	Plain water provided by the race	Sports drinks provided by the race	Other	FPR	NPT	NPS	Other
Total No. (%)								
	234(50.8%)	237(51.4%)	252(54.7%)	13(2.8%)	356(79.8%)	40(8.7%)	40(8.7%)	10(2.2%)
Gender No. (%)								
Male	185(32.4%)	181(31.7%)	193(33.8%)	12(2.1%)	281(81.2%)	26(7.5%)	31(9.0%)	8(2.3%)
Female	49(29.7%)	56(33.9%)	59(35.8%)	1(0.6%)	75(75.0%)	14(14.0%)	9(9.0%)	2(2.0%)
*p*	0.398	0.572	0.630	0.193	0.173	0.046	0.990	0.853
Age No. (%)								
18–34 years	72(50.7%)b	67(47.2%)	83(58.5%)	5(3.5%)	101(74.8%)	14(10.4%)	18(13.3%)	2(1.5%)
35–44 years	101(52.6%)	105(54.7%)	94(49.0%)	3(1.6%)	155(83.8%)	11(5.9%)	15(8.1%)	4(2.2%)
45–54 years	54(53.5%)	51(50.5%)	60(59.4%)	4(4 0.0%)	81(81.0%)	12(12.0%)	5(5.0%)	2(2.0%)
55–64 years	7(26.9%)	14(53.8%)	15(57.7%)	1(3.8%)	19(73.1%)	3(11.5%)	2(7.7%)	2(7.7%)
*p*	0.091	0.586	0.225	0.585	0.193	0.292	0.151	0.271
Performance levels No. (%)								
Public elite	39(54.2%)	41(56.9%)	40(55.6%)	4(5.6%)	57(81.4%)^a^	7(10.0%)	3(4.3%)	3(4.3%)
Public Level 1	69(53.9%)	61(47.7%)	78(60.9%)	4(3.1%)	106(83.5%)	11(8.7%)	10(7.9%)	(0.0%)
Public Level 2	79(46.7%)	96(56.8%)	95(56.2%)	4(2.4%)	135(81.8%)	10(6.1%)	16(9.7%)	4(2.4%)
Not certified	47(51.1%)	39(42.4%)	39(42.4%)	1(1.1%)	58(69.0%)	12(14.3%)	11(13.1%)	3(3.6%)
*p*	0.586	0.087	0.050	0.370	0.055	0.193	0.270	0.176

^a^*p* = 0.003 vs. “Not Certified” (within group); ^b^*p* = 0.032 vs. “55–64 years” (within group). FPR means follow the personal rehydration plan made before the race and refill at the rehydration stations according to the set number of kilometers; NPT means have no rehydration plan and refill when they feel thirsty; NPS means no rehydration plan, drink wherever there is a water station.

Throughout the race cycle, 50.8% of participants chose to bring their own sports supplements, 51.4% chose to drink event-provided plain water, 54.7% chose event-provided beverages, and 2.8% chose other sources of rehydration. Among participants of different ages, a significantly higher percentage of participants under 34 years old brought their own fluids than those over 55 years of age (*p* = 0.032) (Table 5).

### 3.4 Knowledge and attitudes related to rehydration

**Figure 4:**
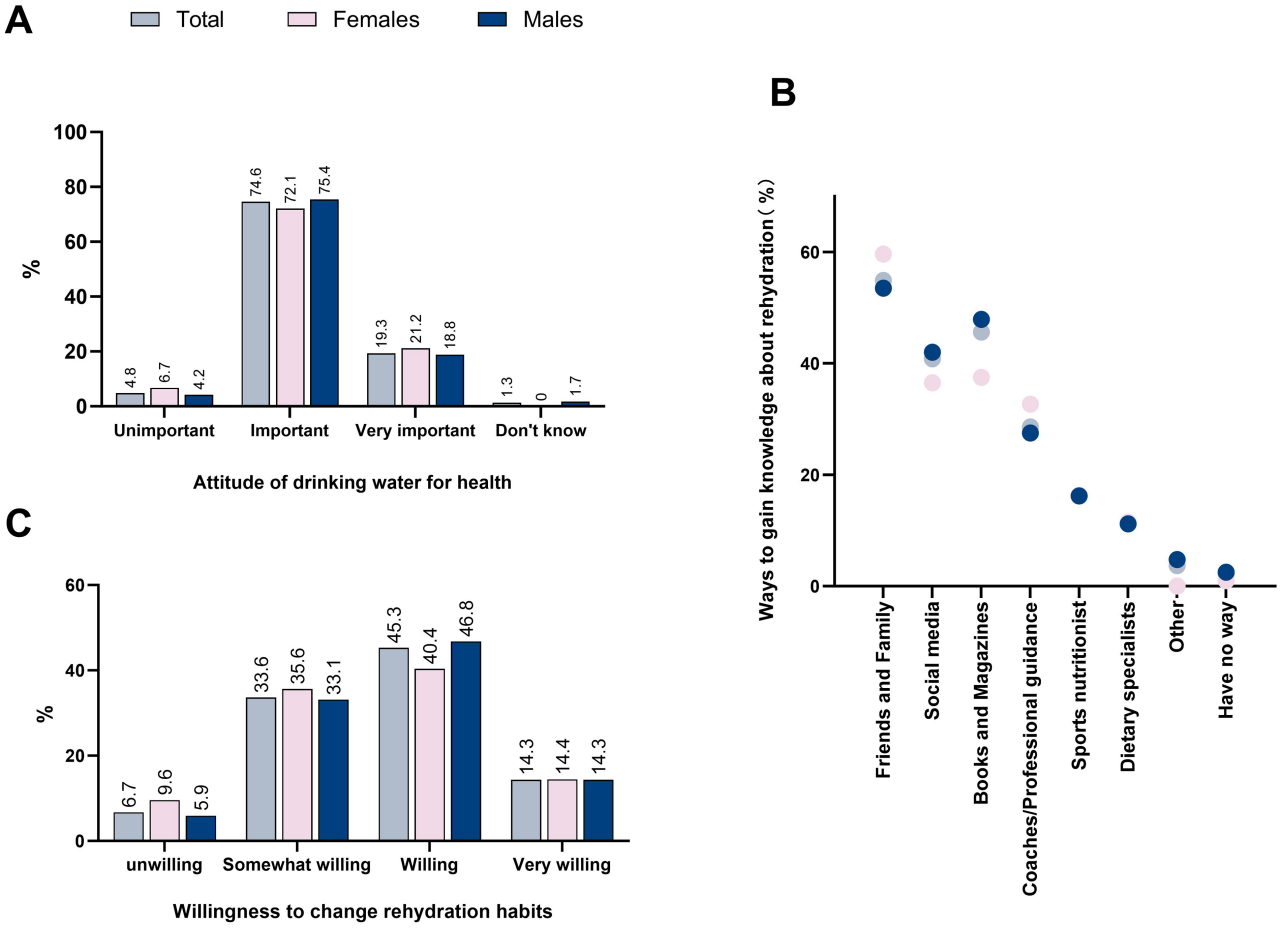
Gender differences in attitudes toward healthy drinking, sources of rehydration knowledge, and willingness to change rehydration behaviors among recreational marathon runners in China **(A)** Attitudinal distribution of the importance of healthy drinking among recreational marathon runners of different genders; **(B)** Distribution of main sources for gaining rehydration knowledge among runners of different genders; **(C)** Distribution of willingness to change rehydration habits among runners of different genders.

One of the primary objectives of this study’s survey was to understand the hydration behavioral habits of Chinese recreational marathon runners and to assess their knowledge about hydration. Through the survey this study found that participants revealed a limited understanding of the standard daily water intake of 1,500–1700 mL (21.5% of participants answered correctly), the difference between mineral spring water, mineral water, and purified water (39.9%), rehydration after exercise (48.8%), and the color of healthy urine (53.1%) (Figure 4B). However, they seemed to be more aware of the need for rehydration before exercise (65.1%), the difference between hydration and rehydration (66.8%), the need for rehydration during exercise (71.1%), the fact that the body is already dehydrated when one is thirsty (78.1%), and the way to drink water (82.6%). Female participants had a better understanding of ways to drink water (84.6%), the difference between mineral spring water, mineral water and purified water (43.3%) and rehydration during exercise (70.4%), whereas male runners had a better understanding of the signals that their body is dehydrated when they are thirsty (78.7%), the difference between water supplementation and fluid replacement (67.5%), before exercise (66.7%) and after exercise (50.4%). Across ages, it appeared to be more difficult to recognize the difference between hydration and rehydration as age increased (*p* < 0.001). In addition, runners with higher levels were more likely to recognize thirst as a sign that the body had become dehydrated (*p* = 0.016) (Table 6).

**Table 6:** Comparison of hydration knowledge accuracy among Chinese recreational marathon runners.

Index	Knows the recommended daily water intake	Understands the correct methods of fluid replacement	Knows the difference between mineral water, electrolyte water, and purified water	Understands that “thirst indicates the body is already dehydrated”	Understands the difference between water supplementation and fluid replacement	Knows the color of healthy urine	Knows the appropriate hydration stages during exercise		
							Pre-exercise	During exercise	Post-exercise
Total No. (%)									
	99(21.5%)	381(82.6%)	184(39.9%)	360(78.1%)	308(66.8%)	245(53.1%)	300(65.1%)	328(71.1%)	225(48.8%)
Gender No. (%)									
Male	77(21.6%)	293(82.1%)	139(38.9%)	281(78.7%)	241(67.5%)	186(52.1%)	238(66.7%)	251(70.3%)	180(50.4%)
Female	22(21.2%)	88(84.6%)	45(43.3%)	79(76.0%)	67(64.4%)	59(56.7%)	62(59.6%)	77(74.0%)	45(43.3%)
*p*	0.928	0.547	0.427	0.551	0.557	0.405	0.184	0.460	0.199
Age No. (%)									
18–34 years	37(26.1%)	110(77.5%)	53(37.3%)	106(74.6%)	102(71.8%)	81(57.0%)	103(72.5%)	90(63.4%)	72(50.7%)
35–44 years	32(16.7%)	161(83.9%)	73(38.0%)	149(77.6%)	135(70.3%)	91(47.4%)	122(63.5%)	135(70.3%)	91(47.4%)
45–54 years	21(20.8%)	87(86.1%)	44(43.6%)	82(81.2%)	61(60.4%)	58(57.4%)	63(62.4%)	80(79.2%)	50(49.5%)
55–64 years	9(34.6%)	23(88.5%)	14(53.8%)	23(88.5%)	10(38.5%)^b^	15(57.7%)	12(46.2%)	23(88.5%)	12(46.2%)
*p*	0.069	0.228	0.341	0.360	0.003	0.991	0.044	0.011	0.930
Performance levels No. (%)									
Public elite	16(22.2%)	61(84.7%)	30(41.7%)	61(84.7%)^a^	46(63.9%)	49(50.0%)	49(68.1%)	50(69.4%)	36(50.0%)
Public Level 1	30(23.4%)	105(82.0%)	55(43.0%)	103(80.5%)	79(61.7%)	8(55.5%)	84(65.6%)	100(78.1%)	68(53.1%)
Public Level 2	37(21.9%)	144(85.2%)	61(36.1%)	133(78.7%)	121(71.6%)	170(52.7%)	112(66.3%)	118(69.8%)	74(43.8%)
Not certified	16(17.4%)	71(77.2%)	38(41.3%)	63(68.5%)	62(67.4%)	67(53.3%)	55(59.8%)	60(65.2%)	47(51.1%)
*p*	0.743	0.400	0.579	0.064	0.316	0.901	0.673	0.182	0.409

^a^*p* = 0.016 vs. “Not Certified” (within group); ^b^*p* <0.001 vs. the “18–34 years” and “35–44 years”(within group).

When asked about the perceived importance of drinking water for health, 93.9% of participants acknowledged its significance (Table 7), with 19.3% rating it as “very important.” Conversely, 4.8% considered it “not important” (Figure 4A). Regarding the willingness to modify hydration practices to enhance athletic performance, 93.3% of participants expressed a willingness to make changes, as shown in Table 7. Among them, 14.3% indicated they were “very willing,” while 6.7% reported being unwilling to change their hydration behavior (Figure 4B).

**Table 7:** Comparison of hydration-related attitudes among Chinese recreational marathon runners.

Index	Acknowledges the importance of water intake for health	Willing to modify hydration habits to enhance athletic performance	Sources of hydration Knowledge							
			Friends and family	Books and magazines	Social media	Coach/ Professional fitness instructor	Sports nutritionist	Dietitian/ Nutrition expert	Other	None
Total No. (%)										
	433(93.9%)	430(93.3%)	253(54.9%)	188(40.8%)	210(45.6%)	132(28.6%)	75(16.3%)	52(11.3%)	17(3.7%)	10(2.2%)
*p*	<0.001	<0.001	—	—	—	—	—	—	—	—
Gender No. (%)										
Male	336(94.1%)	336(94.1%)	191(53.5%)	150(42.0%)	171(47.9%)	98(27.5%)	58(16.2%)	40(11.2%)	17(4.8%)	9(2.5%)
Female	97(93.3%)	94(90.4%)	62(59.6%)	38(36.5%)	39(37.5%)	34(32.7%)	17(16.3%)	12(11.5%)	0(0.0%)	1(1.0%)
*p*	0.750	0.181	0.270	0.317	0.061	0.298	0.981	0.925	0.023	0.337
Age No. (%)										
18–34 years	132(93.0%)	132(93.0%)	83(58.5%)	57(40.1%)	59(41.5%)	30(21.1%)	16(11.3%)	18(12.7%)	4(2.8%)	5(3.5%)
35–44 years	181(94.3%)	178(92.7%)	108(56.3%)	81(42.2%)	91(47.4%)	58(30.2%)	33(17.2%)	16(8.3%)	5(2.6%)	4(2.1%)
45–54 years	94(93.1%)	94(93.1%)	52(51.5%)	37(36.6%)	47(46.5%)	36(35.6%)	22(21.8%)	13(12.9%)	3(3.0%)	1(1.0%)
55–64 years	26(100.0%)	26(100.0%)	10(38.5%)	13(50.0%)	13(50.0%)	8(30.8%)	4(15.4%)	5(19.2%)	5(19.2%)	0(0.0%)
*p*	0.555	0.572	0.243	0.612	0.699	0.084	0.172	0.279	<0.001	0.481
Performance levels No. (%)										
Public elite	66(91.7%)	65(90.3%)	36(50.0%)	24(33.3%)	31(43.1%)	25(34.7%)^a^	17(23.6%)	5(6.9%)	6(8.3%)	0(0.0%)
Public Level 1	123(96.1%)	122(95.3%)	69(53.9%)	53(41.4%)	59(46.1%)	43(33.6%)	25(19.5%)	20(15.6%)	4(3.1%)	2(1.6%)
Public Level 2	159(94.1%)	158(93.5%)	93(55.0%)	77(45.6%)	84(49.7%)	46(27.2%)	21(12.4%)	16(9.5%)	3(1.8%)	6(3.6%)
Not certified	85(92.4%)	85(92.4%)	55(59.8%)	34(37.0%)	36(39.1%)	18(19.6%)	12(13.0%)	11(12.0%)	4(4.3%)	2(2.2%)
*p*	0.555	0.571	0.651	0.280	0.407	0.082	0.094	0.225	0.096	0.342

^a^*p* = 0.029 vs. “Not Certified” (within group).

The reliability of knowledge related to fluid replenishment came from coaches (28.6%), sports dietitians (16.3%), and dietary experts (11.3%). However, these specialized sources were consulted less frequently than informal sources, such as family and friends (54.9%), social media (45.6%), or books and magazines (40.8%), with males appearing to be more inclined to obtain rehydration knowledge from social media than females. A small number of participants, mainly at the “Public Elite” level, preferred to obtain knowledge from professional sources such as coaches (*p* = 0.029) (Table 7).

## 4 Discussion

Optimal hydration is critical for maintaining homeostatic regulation of organ systems and biochemical equilibrium (31). However, this study found that Chinese recreational marathon runners were generally under-hydrated. Based on the standard of 1,500 mL to 1700 mL of water per day for normal adults, as recommended in the Dietary Guidelines for Chinese Residents (2022) (30), only 27.5% of participants were able to meet this minimum threshold. This rate is notably lower than the 67.6% reported by Zuo et al. (32) for cities such as Beijing, Shanghai, Guangzhou, and Chengdu, or the 60% reported by Malisova and Athanasatou et al. (33) in European countries, and by Shaheen et al. (34), who noted that the water intake of populations in the Middle East met the recommended standard. However, it is similar to the attainment rates reported by Martinez et al. (35) for Mexico (10–25%) and Brazil (21%). The achievement of the daily water intake standard varied across studies, potentially due to differences in external factors (e.g., geographic characteristics, climatic conditions), sociocultural context (e.g., dietary habits, ethnic lifestyles), and study design characteristics (e.g., sample size, age group stratification). Particularly concerning is the observation that 22.3% of participants had a daily water intake of less than 800 mL. Given the impact of exercise and heat stress on body water homeostasis, and referencing foreign studies indicating higher water intake among individuals with elevated exercise levels (36–38), it is hypothesized that Chinese recreational marathon participants may experience chronic mild dehydration for extended periods or may even be dehydrated before training. This variability underscores the need for education among Chinese recreational marathon runners regarding rehydration to enhance their understanding and help mitigate health risks. Furthermore, this study revealed that the proportion of insufficient water intake was significantly higher among” Not Certified” runners compared to “Public Elite” runners. This discrepancy may reflect differences in daily training mileage or hydration awareness among performance levels. Collectively, the results suggest that while hydration knowledge and practices may improve with athletic performance level, overall water intake among Chinese recreational marathon runners remains inadequate.

An accurate assessment of an individual’s hydration status is a fundamental component of a scientific rehydration strategy. The presence of dehydration can typically be identified through thirst signals and urine color, with the latter serving as a simple yet effective indicator of daily hydration status (39). However, the present study revealed that 54.9% of participants relied on thirst as their primary indicator of hydration status, and 46.9% were unaware of the urine color that corresponds to adequate hydration. Notably, a higher proportion of participants under the age of 34 reported using thirst signals as their primary cue for hydration. The physiological phenomenon of lagged thirst may contribute to a detrimental cycle in which individuals experience dehydration, fail to adequately rehydrate, and subsequently exacerbate their dehydration, particularly during physical exertion (40, 41). This cycle increases the risk of fluid and electrolyte imbalance, especially among those who train frequently. These findings suggest that Chinese recreational marathon runners generally lack scientific understanding of fluid replacement and hold misconceptions regarding accurate self-assessment of hydration status, which may contribute to their insufficient daily fluid intake. Despite some degree of awareness, only 27.5% of participants met the recommended daily water intake, reflecting a disconnect between perceived hydration awareness and actual fluid consumption. This further amplifies the risk of chronic, subclinical dehydration. The general neglect of dehydration risks, limited self-regulated fluid intake, and poor monitoring of hydration biomarkers, particularly urine color, which can be easily observed visually, highlight the urgent need to integrate biomarker-based education into public hydration strategies. Therefore, future efforts should prioritize the widespread dissemination of fluid replacement knowledge among Chinese recreational marathon runners, with a focus on improving daily hydration practices and fostering greater awareness of the critical link between proper hydration, athletic performance, and long-term physical health.

Scientific hydration strategies are essential for maintaining physiological function during marathon exercise. Prolonged high-intensity running relies heavily on evaporative heat loss to maintain core temperature homeostasis (17, 42), during which substantial water and electrolyte losses are inevitable. Numerous international studies have demonstrated that inappropriate rehydration behavior can compromise both health and athletic performance. On the one hand, excessive intake of pure water dilutes body water, accompanied by sweat sodium loss, which can lead to a dilutional decrease. risk of exercise-associated hyponatremia (43); on the other hand, inadequate fluid intake may impair thermoregulation and cardiovascular function, heightening the risk of heat stroke, exertional rhabdomyolysis, and a marked reduction in aerobic capacity (44), especially in endurance athletes.

In this study, we examined the rehydration behaviors of Chinese recreational marathon runners across three critical stages—before, during, and after competition—and identified widespread misconceptions regarding appropriate hydration practices. The core issues centered on the persistent risk of inadequate fluid intake and the general lack of scientific rehydration strategies. According to recommendations by the American College of Sports Medicine (ACSM), sodium-containing fluids should be consumed at a volume of 5–10 mL/kg approximately 2 h prior to competition to optimize hydration status (45, 46). However, 57.7% of the participants in our study reported consuming only 150 mL of fluids prior to the race, and the majority selected plain water as their primary source of hydration. This combination of insufficient volume and electrolyte deficiency was not confined to any particular subgroup, suggesting that a large proportion of recreational runners may begin competition in a state of pre-existing dehydration. These findings are consistent with previous reports by Maughan and McCrink et al. (47, 48), who observed that many athletes were already dehydrated prior to exercise. Collectively, the results reinforce our hypothesis that Chinese recreational marathon runners may experience chronic mild dehydration, and in some cases, may remain persistently dehydrated throughout the duration of the race.

Fluid replacement during exercise is calculated based on exercise intensity and sweat rate (49). Noakes and Martin et al. (50) recommend that athletes aim to consume 400–800 mL of fluid per hour during endurance activities. However, the present study found that many Chinese recreational marathon runners consumed only 150 mL of fluid during the race. This suggests that nearly half of these runners may experience dehydration during competition. This phenomenon may be attributed to concerns over gastrointestinal discomfort caused by fluid intake during exercise (51). Therefore, it is recommended that runners develop individualized hydration strategies aligned with their physiological status, training level, environmental conditions, and event-specific demands. To improve gastrointestinal tolerance to fluid intake, athletes should also engage in periodic gut training during exercise. Additionally, fluid consumption should be adjusted according to race duration and environmental heat stress. Notably, the current findings diverge from previous studies reporting higher fluid intake among runners with less marathon experience, slower training paces, or longer race durations (52–54). In contrast, our study observed lower in-race fluid intake among Chinese recreational marathon runners, with no significant variation across performance levels. This indicates a general lack of awareness regarding hydration practices and a greater susceptibility to exercise-induced dehydration in this population. Interestingly, female runners were more likely to select fluid volumes of 750 mL or more during the race, a finding consistent with international studies (43, 52). This may reflect both greater hydration awareness and physiological differences (e.g., smaller body mass and greater concern over fluid loss), but it may also increase the risk of exercise-associated hyponatremia if sodium is not adequately replenished.

Post-exercise fluid replacement is essential for supporting physiological recovery and restoring homeostasis. It is generally recommended that fluids lost during exercise be replenished at a rate of approximately 125–150% of the fluid deficit—typically, 1.25 to 1.5 liters of fluid per 1 kg of body weight lost—alongside appropriate sodium supplementation to restore water-electrolyte balance and maintain systemic fluid homeostasis (55, 56). The present study also found similar behavior to Judge et al. (22), who observed a lack of voluntary hydration among participants following exercise; Nearly half of Chinese recreational marathon runners chose to replenish only 150 mL of fluid after the race. This may be related to factors such as professional rehydration knowledge, biased understanding of scientific advice, and inadequate design of rehydration stations and environments. In addition, there was significant variability in insufficient hydration by performance levels, with runners with high levels of athleticism being able to recognize the role of post-exercise hydration in promoting physiological recovery and being more likely to adopt positive hydration behaviors. These findings are consistent with previous research emphasizing the importance of hydration attitudes in influencing rehydration intake behavior (57).

In this study, we found that a higher proportion of Chinese recreational marathon runners consumed sports drinks during the race (56.6%) compared with the 50% reported by Felder et al. (58). To prevent dehydration and delay the onset of exercise fatigue and “hitting the wall” (59), it is necessary to consume a certain proportion of carbohydrates and electrolytes during marathon races to promote enhanced fluid absorption and supports sustained exercise performance (46). The nutritional value of plain water alone generally lacks the nutritional components necessary, while the additional sports drink intake can fulfill these needs (60) and provide a convenient option for runners who may not have prepared specialized rehydration fluids in advance. Notably, significant gender differences were observed in post-race consumption of carbonated beverages. Female runners were more likely to consume such drinks, which may reflect a greater preference for sweetness or a form of emotional compensation following strenuous exercise. Women tend to have higher body fat and lower fat-free mass, with elevated leptin levels and a more sensitive ghrelin response. This makes them more likely to consume sweet foods after exercise as a rapid means of restoring energy and achieving psychological comfort (61). Additionally, women’s eating behaviors are more strongly influenced by emotions, and the psychological association between sweet foods and emotional consolation may further reinforce their preference for high-sugar foods. However, the high-sugar, low-sodium nature of carbonated beverages may interfere with water absorption and exacerbate electrolyte loss and the CO₂ gas released there may also exert greater stress on the gastrointestinal lining (62), causing greater renal oxidative stress and mild renal injury, especially after ingestion of fructose-rich beverages in a dehydrated state (63). Despite these potential risks, 15.6% of participants still chose carbonated beverages post-race, likely due to the psychological satisfaction derived from their distinctive flavor and sensory stimulation.

Data on sources and strategies of rehydration during the race reflect the diversity and variability in rehydration behaviors among recreational marathon runners in China. The survey revealed that some participants did not follow a structured rehydration plan and instead relied on thirst cues during the race. Similar patterns have been reported in previous studies (19, 22, 64, 65), especially among females and the “Not Certified” participants. A key contributing factor to this phenomenon is insufficient knowledge regarding hydration. The present study confirmed that runners with higher performance levels were significantly more likely to formulate a pre-race hydration plan (66). In addition, health-related concerns were identified as major motivational drivers for behavioral change. A substantial majority of participants (95.2%) acknowledged the importance of hydration for maintaining health, and 93.3% expressed a willingness to modify their hydration practices to enhance performance. These findings are consistent with prior research indicating that health awareness and performance goals jointly motivate positive behavioral adaptations (20, 23). Collectively, these results underscore the presence of outcome-oriented behavioral change tendencies among Chinese recreational marathon runners and highlight the urgent need for targeted educational interventions aimed at promoting scientifically informed hydration practices.

Based on the correct responses to the participants’ knowledge of rehydration, it was found that their understanding of the field of scientific rehydration was low. The results of this study were similar to those of Chi et al. (67). Only 21.5% of the respondents were aware of the recommended daily water intake. Assessing an individual’s hydration status is also a key element of a scientific rehydration strategy. Urine color is a simple and effective indicator for evaluating daily hydration status (39). The survey showed that only 53.1% of Chinese recreational marathon runners could correctly identify a healthy urine color. The findings of the present study confirm those reported by Rosenbloom et al. (68) and Spokely et al. (69), indicating a widespread knowledge gap in hydration across the general population, making it difficult for individuals to maintain adequate hydration. Overall, the low level of hydration knowledge among Chinese recreational marathon runners highlights the urgency of enhancing health education and incorporating biomarker monitoring into hydration education.

The present investigation found that family and friends (54.9%), social media (45.6%), and books and magazines (40.8%) were the main sources of knowledge about rehydration for runners, but the quality of information from these sources varied, which may lead to runners obtaining incomplete or incorrect health knowledge. In contrast to Nieper’s (70) study, in which 75.0% of athletes had access to a sports dietitian, only a minority of participants in this study had access to a sports dietitian (16.3%) or a coach (28.6%). This highlights the underrepresentation of professionals in mass fitness; one contributing factor may be the free and unsystematic nature of the participants’ training routines. On the other hand, the services of sports dietitians and professional coaches may be constrained by factors such as geographical limitations and cost, making it difficult for some runners to obtain professional guidance. Therefore, it is recommended to strengthen the knowledge, attitudes, and education related to scientific fluid replenishment among Chinese recreational marathon runners, to help this population recognize the close relationship between hydration, athletic performance, and physical health, ultimately aiming to promote physical well-being and enhance athletic capacity.

## 5 Limitations, implications, and future directions

Although this study systematically evaluated the hydration knowledge, attitudes, and behaviors of Chinese recreational marathon runners, certain limitations remain. First, as a cross-sectional design was employed, causal relationships between hydration behaviors and health outcomes cannot be established. Second, the data were primarily based on self-reported information, which may be subject to recall bias or social desirability bias—particularly in measures such as daily fluid intake and hydration practices—potentially resulting in discrepancies from actual behavior. Third, although the sample included participants from various regions and events, there was some imbalance in geographic and age distribution, which may limit the generalizability of the findings.

Nevertheless, this study represents the first nationwide systematic investigation of hydration practices among recreational marathon runners in China. It identified widespread issues such as insufficient daily fluid intake, inadequate hydration during race periods, and limited knowledge levels. These findings provide empirical evidence for the development of targeted health education strategies aimed at improving hydration behaviors within this population.

Future research should explore the unique characteristics of the Chinese population in terms of body composition, physiological parameters, and lifestyle, in order to develop hydration strategies and intake guidelines that are better tailored to the specific needs of East Asian populations. Specifically, future studies should focus on key parameters such as fluid type, hydration volume, and electrolyte concentration to construct individualized hydration plans that are both targeted and scientifically sound, meeting the physiological demands of different populations under varying environmental conditions and exercise intensities. In addition, further research may be needed to specifically examine the relationship between athletic performance level and fluid intake. Meanwhile, emphasis should be placed on building self-monitoring capabilities for hydration status, shifting runners from passive to active hydration management. It is recommended to integrate modern wearable devices and digital health tools to develop self-assessment scales or intelligent systems based on indicators such as urine color, body weight change, and sweating rate, thereby enabling runners to dynamically evaluate their hydration status and receive behavioral feedback to improve the proactivity, scientific rigor, and timeliness of their hydration practices.

## 6 Conclusion

In summary, the results of this research study suggest that daily underhydration is very common among Chinese recreational marathon runners (72.5%), and that underhydration behaviors during races are even more pronounced and not restricted to a specific subgroup. A lack of knowledge about hydration is the main reason for this phenomenon. These findings highlight the need to educate this population with scientific knowledge on proper hydration.

Meanwhile, the findings of this study also provide empirical evidence to inform both race organizers and individual athletes in developing targeted intervention strategies. It is recommended that recreational marathon runners enhance their awareness and behavioral capacity regarding scientific hydration management. Particularly during routine training, greater emphasis should be placed on the dynamic assessment of individual hydration status. For example, simple indicators such as urine color and body weight changes can be used to identify potential dehydration risks in a timely manner, thereby gradually fostering proactive and evidence-based hydration habits.

In races of varying distances, the density and placement of hydration stations, the types of beverages provided, and the delivery methods should all be scientifically tailored according to real-time meteorological conditions on race day—such as temperature, and humidity—as well as racecourse characteristics, including elevation profile, shading, and terrain complexity, in order to optimize fluid replacement efficiency.

## Data Availability

The original contributions presented in the study are included in the article/Supplementary material, further inquiries can be directed to the corresponding author/s.
