# Supplementary material for: Hydration behaviors, knowledge, and attitudes among Chinese recreational marathon runners: a cross-sectional study
Source: Front Nutr. 2025 Aug 13;12:1621966. doi: 10.3389/fnut.2025.1621966 (PMC12380549; doi:10.3389/fnut.2025.1621966)
Supplement: Supplementary file 1 [file Table_1.docx]

Supplementary Material

# Supplementary Figures


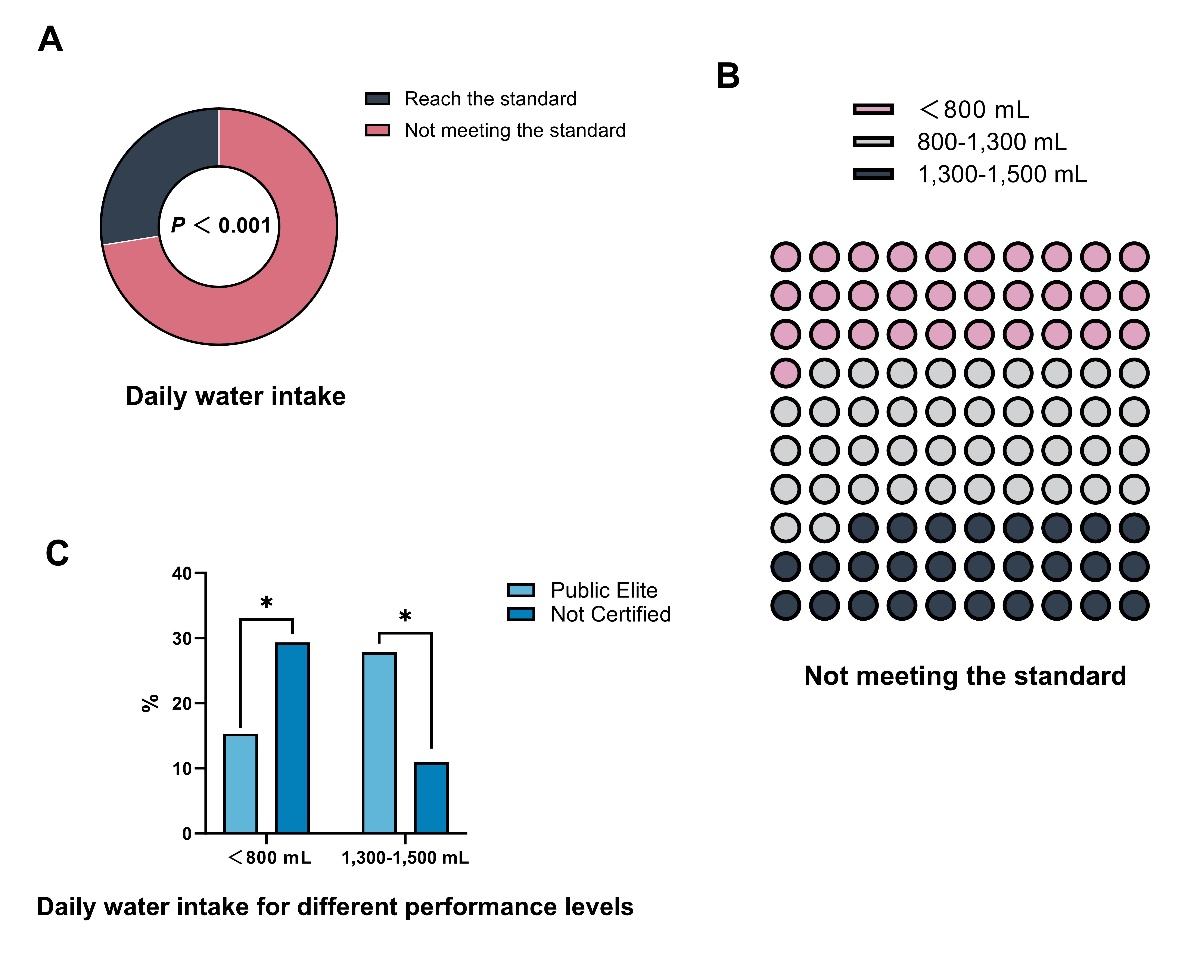


**Supplementary Figure 1.** Daily water intake status and subgroup comparisons among Chinese recreational marathon runners. (A) Proportion of participants who met (dark gray) versus did not meet (pink) the recommended daily water intake. The difference was statistically significant (*P* < 0.001). (B) Distribution of daily water intake levels among participants who did not meet the standard. Intake was categorized as <800 mL (pink), 800–1,300 mL (gray), and 1,300–1,500 mL (dark blue). (C) Comparison of daily water intake between “Public Elite” runners and “Not certified” runners. A higher proportion of “Public Elite” runners consumed 1,300–1,500 mL daily, while a greater proportion of “Not certified” runners consumed <800 mL. **P* < 0.05.

**
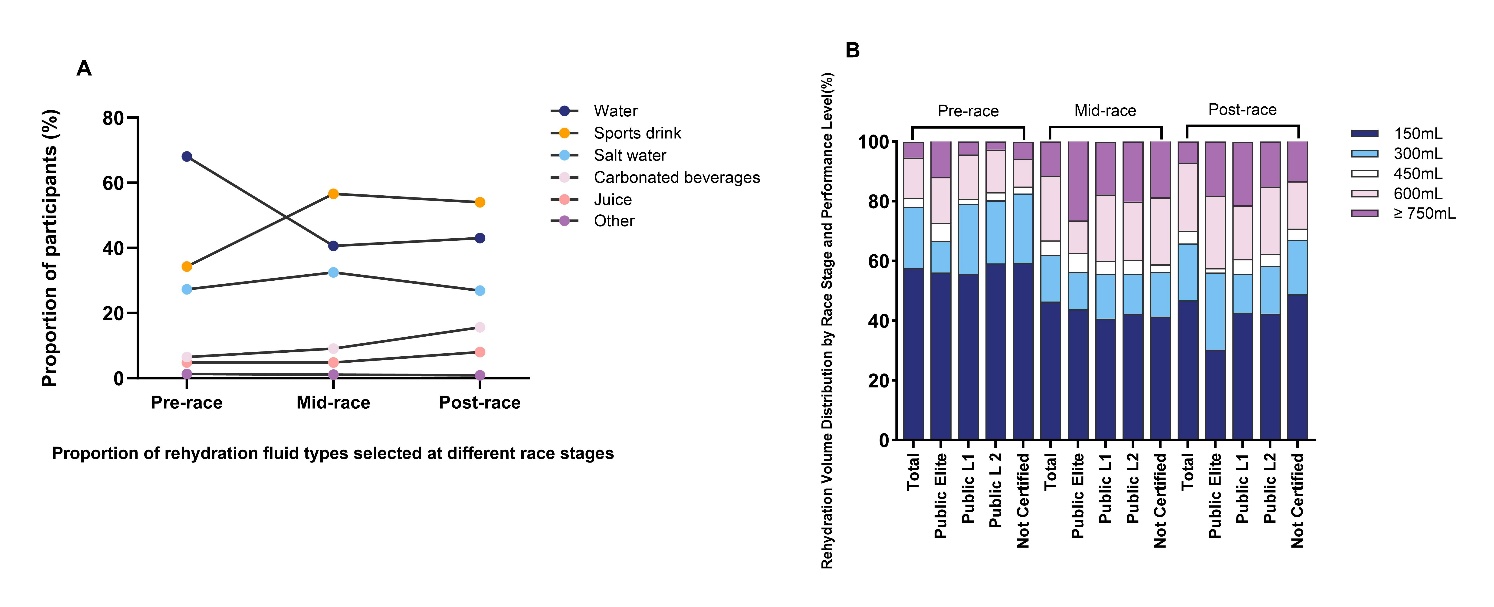
**

**Supplementary Figure 2.** Types and volumes of fluid replacement during training and competition among recreational marathon runners. (A) Distribution of Fluid Type Preferences Among Runners During the Race; (B) Comparison of In-Race Fluid Intake Among Runners of Different Performance Levels.

**
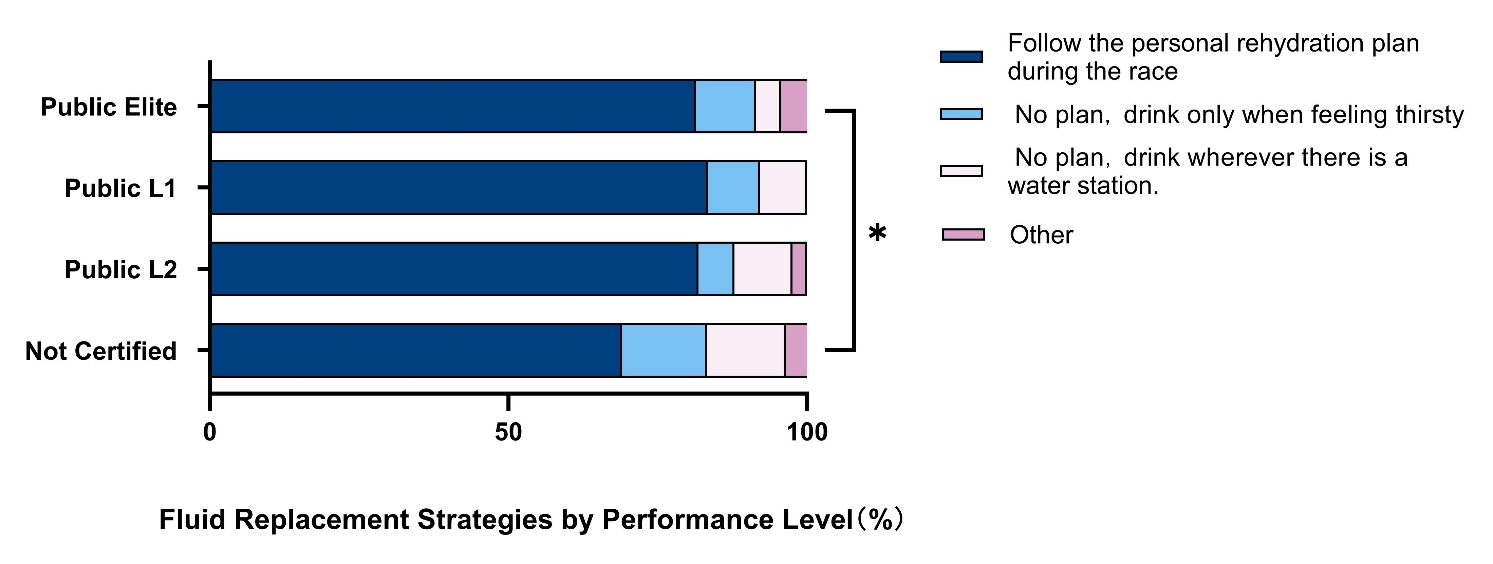
**

**Supplementary Figure 3.** Distribution of Fluid Replacement Strategies during the Race across Different Performance Levels among Recreational Marathon Runners. (*)Indicates that there is a significant difference in “Not Certified” compared to other performance levels (p = 0.003); **FPR** means follow the personal rehydration plan made before the race and refill at the rehydration stations according to the set number of kilometers; **NPT** means have no rehydration plan and refill when they feel thirsty; **NPS** means no rehydration plan, drink wherever there is a water station.

**
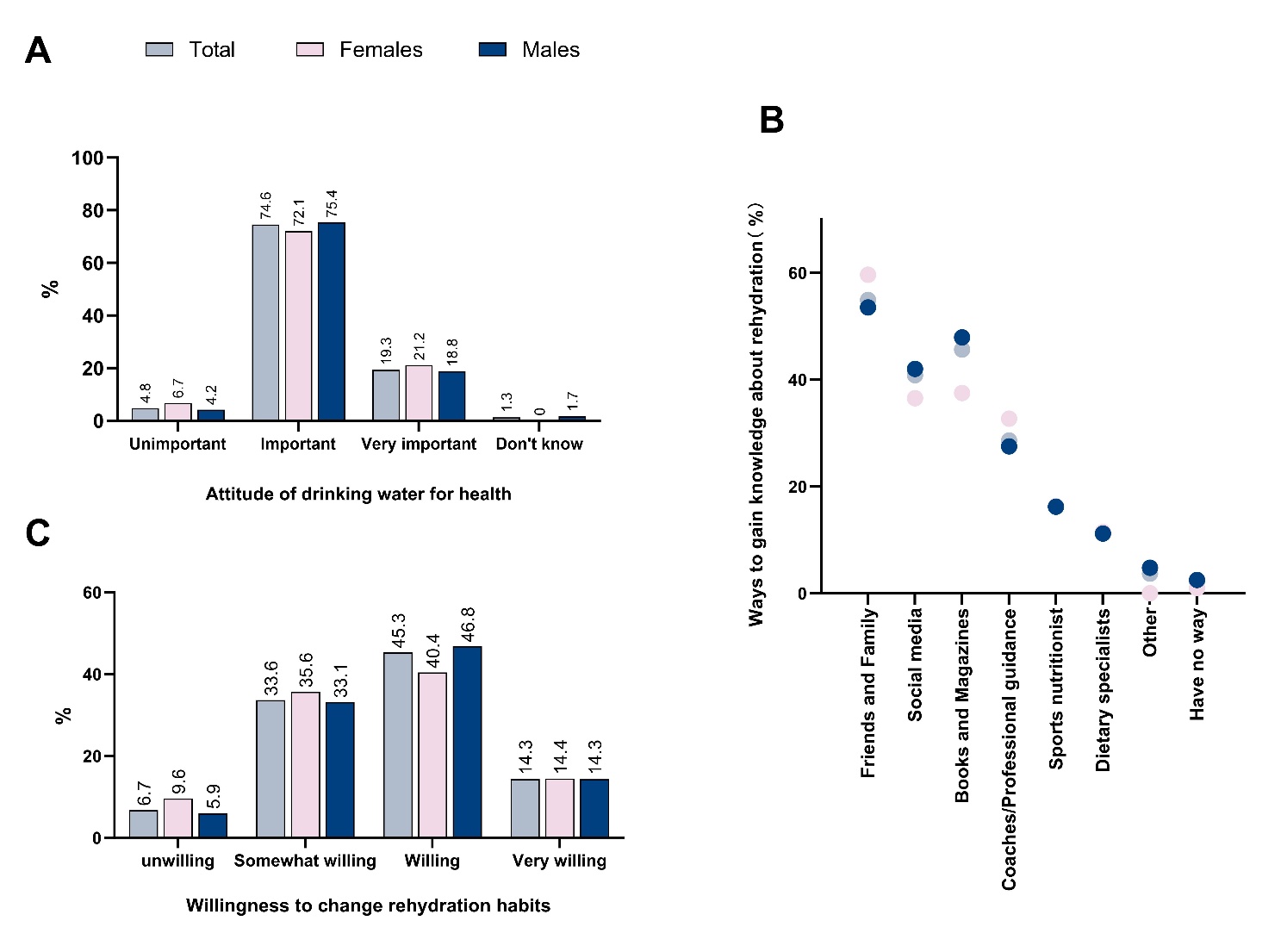
**

**Supplementary Figure 4.** Gender Differences in Attitudes Toward Healthy Drinking, Sources of Rehydration Knowledge, and Willingness to Change Rehydration Behaviors among Recreational Marathon Runners in China (A) Attitudinal Distribution of the Importance of Healthy Drinking among Recreational Marathon Runners of Different Genders; (B) Distribution of Main Sources for Gaining Rehydration Knowledge among Runners of Different Genders; (C) Distribution of Willingness to Change Rehydration Habits among Runners of Different Genders.

1. **Supplementary Table**

**Supplementary Table 1.** Athletic Performance Classification Standards for Amateur Marathon Runners in China.

| **Event** | **Group** | **Level** | **Under 34** | **35–39 yrs** | **40–44 yrs** | **45–49 yrs** | **50–54 yrs** | **55–59 yrs** | **60–64 yrs** |
| --- | --- | --- | --- | --- | --- | --- | --- | --- | --- |
| **Marathon** | Male | Public Elite | 3:00:00 | 3:10:00 | 3:15:00 | 3:20:00 | 3:30:00 | 3:40:00 | 3:50:00 |
|  |  | Public Level 1 | 3:30:00 | 3:40:00 | 3:45:00 | 3:50:00 | 4:00:00 | 4:10:00 | 4:20:00 |
|  |  | Public Level 2 | 4:00:00 | 4:10:00 | 4:15:00 | 4:20:00 | 4:30:00 | 4:40:00 | 4:50:00 |
|  | Female | Public Elite | 3:20:00 | 3:30:00 | 3:35:00 | 3:40:00 | 3:50:00 | 4:00:00 | 4:20:00 |
|  |  | Public Level 1 | 3:50:00 | 4:00:00 | 4:05:00 | 4:10:00 | 4:20:00 | 4:30:00 | 4:50:00 |
|  |  | Public Level 2 | 4:20:00 | 4:30:00 | 4:35:00 | 4:40:00 | 4:50:00 | 5:00:00 | 5:20:00 |
| **Half Marathon** | Male | Public Elite | 1:25:00 | 1:30:00 | 1:32:00 | 1:35:00 | 1:40:00 | 1:45:00 | 1:50:00 |
|  |  | Public Level 1 | 1:35:00 | 1:40:00 | 1:42:00 | 1:45:00 | 1:50:00 | 1:55:00 | 2:00:00 |
|  |  | Public Level 2 | 1:55:00 | 2:00:00 | 2:02:00 | 2:05:00 | 2:10:00 | 2:15:00 | 2:20:00 |
|  | Female | Public Elite | 1:35:00 | 1:40:00 | 1:42:00 | 1:45:00 | 1:50:00 | 1:55:00 | 2:05:00 |
|  |  | Public Level 1 | 1:45:00 | 1:50:00 | 1:52:00 | 1:55:00 | 2:00:00 | 2:05:00 | 2:15:00 |
|  |  | Public Level 2 | 2:05:00 | 2:10:00 | 2:12:00 | 2:15:00 | 2:20:00 | 2:25:00 | 2:35:00 |

*The standard, issued by the Chinese Athletics Association, classifies athletic performance into three levels—Public Elite, Public Level 1, and Public Level 1—based on the official finish times of participants in full or half marathons across different age and gender groups.
